# REDUCING ANTIPSYCHOTIC PRESCRIBING IN NURSING HOMES: FACILITATORS AND BARRIERS FOR HIGH-IMPACT STATE INITIATIVES

**DOI:** 10.1093/geroni/igz038.572

**Published:** 2019-11-08

**Authors:** Stephen Crystal, Richard Hermida, Olga F Jarrín, Sheree Neese-Todd, Beth Angell, Marsha Rosenthal

**Affiliations:** 1 Rutgers, The State University of New Jersey, New Brunswick, New Jersey, United States; 2 Rutgers University, New Brunswick, New Jersey, United States; 3 Virginia Commonwealth University, Richmond, Virginia, United States

## Abstract

In conjunction with the National Partnership to Improve Dementia Care in Nursing Homes initiated in 2012, states implemented initiatives to reduce antipsychotic use. All achieved substantial reductions, but improvement varied across states. By 2018, several states had achieved reductions of more than 45%, including several of the largest states. These reductions are noteworthy given the challenging nature of behavioral symptoms of dementia, and difficulties encountered historically and internationally in changing strongly-rooted clinical practices. How were these successful interventions achieved in high-performing state initiatives? What were the barriers encountered and facilitators that helped overcome these barriers? What does this experience suggest for sustainability of change? To address these questions, we draw on a mixed-methods study of antipsychotic prescribing in nursing homes incorporating analyses of prescribing data, state policy case studies, and facility case studies. Successful states integrated large-scale educational initiatives with strong regulatory action, often focusing especially on laggard facilities. Texas’ initiative was particularly noteworthy, achieving a 56.5% reduction across its far-flung network of nearly 100,000 residents and 1,200 facilities. Texas used metrics to identify facilities that achieved notable reductions in antipsychotic prescribing, and encouraged them to share their strategies with “late adopters”. The state deployed a designated Quality Monitoring Program (QMP), distinct from the survey process, to provide on-site technical assistance to laggard facilities, and provided education for all levels of staff and assistance in implementing data-driven improvement strategies. Successful state initiatives achieved considerable buy-in on the need to reduce antipsychotic use, a key factor in achieving successful system change.

